# Design and analysis of adaptive Super-Twisting sliding mode control for a microgyroscope

**DOI:** 10.1371/journal.pone.0189457

**Published:** 2018-01-03

**Authors:** Zhilin Feng, Juntao Fei

**Affiliations:** College of IoT Engineering, Hohai University, Changzhou, China; Lanzhou University of Technology, CHINA

## Abstract

This paper proposes a novel adaptive Super-Twisting sliding mode control for a microgyroscope under unknown model uncertainties and external disturbances. In order to improve the convergence rate of reaching the sliding surface and the accuracy of regulating and trajectory tracking, a high order Super-Twisting sliding mode control strategy is employed, which not only can combine the advantages of the traditional sliding mode control with the Super-Twisting sliding mode control, but also guarantee that the designed control system can reach the sliding surface and equilibrium point in a shorter finite time from any initial state and avoid chattering problems. In consideration of unknown parameters of micro gyroscope system, an adaptive algorithm based on Lyapunov stability theory is designed to estimate the unknown parameters and angular velocity of microgyroscope. Finally, the effectiveness of the proposed scheme is demonstrated by simulation results. The comparative study between adaptive Super-Twisting sliding mode control and conventional sliding mode control demonstrate the superiority of the proposed method.

## Introduction

Microgyroscope is a basic measurement element of inertial navigation and guidance system. Because of its superiority in structure, bulk and price, microgyroscope is widely used in aerospace, navigation, aviation, and consumer electronics [1]. However, its small size poses a challenge on controller design and microfabrication. The effects of temperature and error in the design and manufacture lead to the decrease of the sensitivity and accuracy. The imprecise microfabrication and disturbances result in mechanical coupling terms between two axes, mechanical–thermal noises, and parameter variations in [2–4], which also consequently degrade the performance of the microgyroscope. Compensating the manufacturing errors and measuring angular velocity becomes the main tasks of microgyroscope control, therefore an effective controller is essential for improving the performance of the microgyroscope by compensating for the mechanical imperfections and the disturbances effectively.

During the past few years, a growing attention has been paid to the feedback control system designs for microgyroscope [5–8]. A novel active disturbance rejection control was designed for a MEMS gyroscope in [5]. An adaptive controller was proposed in [6], but it did not fully account for the mechanical coupling terms on the drive axis caused by the manufacture imperfections. A robust adaptive control strategy using a fuzzy compensator for MEMS triaxial gyroscope was discussed in [7]. An adaptive dynamic surface control for MEMS triaxial gyroscope with nonlinear inputs was proposed in [8]. Park et al. [9] designed an adaptive force-balancing control for a MEMS z-axis gyroscope using a trajectory-switching algorithm. In recent years, some kinds of sliding mode control have been developed. Liu et al. [10] designed a global sliding mode controller for chaotic systems. Global robust optimal sliding mode control for uncertain affine nonlinear systems was showed in [11]. Especially, some sliding mode control methods have been applied to microgyroscope systems [12–15].

However, there is chattering if only using sliding mode control and the most representative characteristic of the conventional sliding mode control is that the convergence of system states to the equilibrium point is usually asymptotical but not in a finite time. In order to avoid these disadvantages, a new type of sliding mode control technique called Super-Twisting sliding mode control is developed, which can make the system states reach the equilibrium point in a finite time and weaken the chattering, playing an important role in sliding mode control. It is not only robust to external disturbances and system uncertainties, but also can precisely adjust and track the system. In addition, only information of the output (sliding variable) is required and time derivative of the output is not needed. Therefore, it is a relatively simple control law, the computation burden can be reduced and a wide range of application can be achieved.

In particular, Super-Twisting algorithm proposed in[16–20] that belongs to the family of high order sliding mode (HOSM) controllers represent an interesting option. It also belongs to the second order sliding mode (SOSM) approach that allows for finite-time convergence to zero of not only the sliding variable but its derivative as well. Roughly speaking, Super-Twisting algorithm consists of zeroing the sliding variable and its first time derivative in a finite time, through a continuous control acting discontinuously on its second time derivative, as discussed in [21]. Finite time control method is another effective strategy to improve disturbance rejection performance.[22–24]. In [25], fixed-time leader-following lag consensus problem of second-order multiagent systems with input delay is discussed by a novel nonsingular terminal sliding mode protocol. The presented sliding mode controller can avoid singularity, eliminate chattering, and achieve exact convergence. SOSM is an excellent option to control nonlinear uncertain systems operating in perturbed environment [26]. All SOSM controllers, except for the Super-Twisting algorithm (STA), require the knowledge of the values of the derivatives[27]. So it can not only solve the nonlinear robust stability of the system well, but also converge to the reference in a finite time mentioned in [28], [29] and avoid chattering [30], [31], [32].

According to the aforementioned works, an adaptive Super-Twisting sliding mode control for microgyroscope with unknown parameters and external uncertainty and disturbance is constructed. The main advantages of the proposed methods in this paper can be summarized as follows.

1) The designed controller consists of an equivalent control and a switching control. The equivalent control ensures that the system reaches the sliding surface. The switching control is designed based on the Super-Twisting algorithm, which forces the system to slide along the sliding surface and achieve robustness to model uncertainties and external disturbances.

2) This method adopt the superiority of Super-Twisting sliding mode control, which can avoid the chattering effectively and make output signal to be continuous and chattering free. It is not necessary to obtain the derivative and extreme value of the sliding mode variable. The superior characteristic is that it adopts the advantages of adaptive control, which can identify and estimate the unknown parameters of microgyroscope system on line.

3) The global asymptotic stability can be guaranteed by this method, ensuring that the sliding mode variable and its first derivative can converge to zero in a finite time and the output trajectory can track the reference trajectory accurately and effectively. This, to a great extent, improves the robustness, sensitivity and accuracy of the control system.

The rest of this paper is organized as follows. Firstly, the dynamics of microgyroscope is described, the sliding mode control is briefly introduced, in particular, the difference between traditional sliding mode control and Super-Twisting sliding mode control is introduced and an adaptive Super-Twisting sliding mode controller for microgyroscope system is presented. Next. the simulation results of adaptive Super-Twisting sliding mode control illustrate the effectiveness and feasibility of the presented method. The final section is a summary and conclusion.

## Materials and methods

In this section, the mathematical model of z-axis microgyroscope is described and the difference between traditional sliding mode control and Super-Twisting sliding mode control is introduced, then, in order to solve the trajectory tracking problem of microgyroscope with unknown model uncertainties and external disturbances, a new adaptive Super-Twisting sliding mode control method is proposed in this section.

### Dynamics of microgyroscope

**Fig 1:**
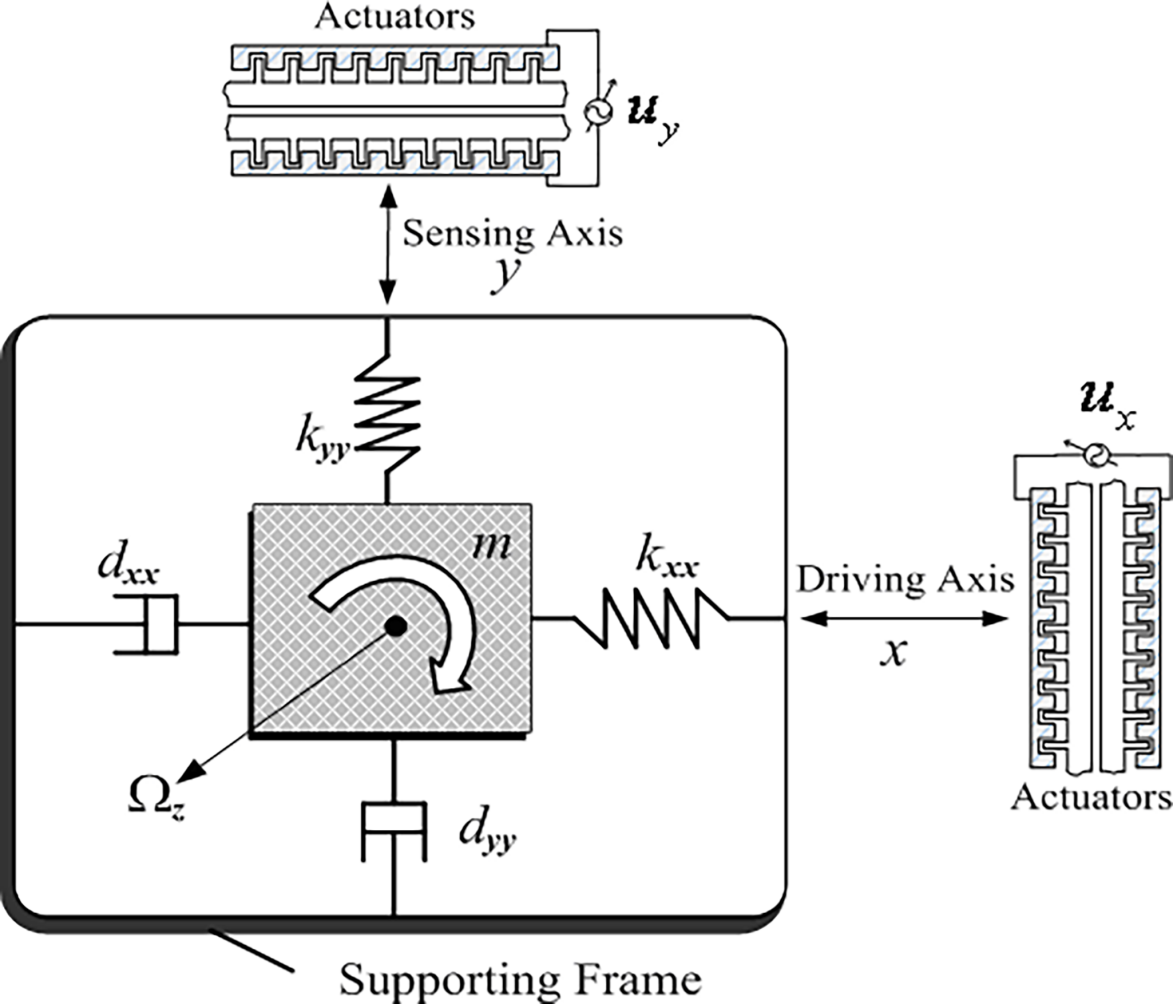
Mass–spring–damper structure of microgyroscopes.

This section mainly introduces the mathematical model of z-axis microgyroscope. Firstly, the dynamics model of the microgyroscope can be simplified into a damped spring mass system. Then the differential equations of the dynamic microgyroscope system will be established. The dynamic differential equation of the microgyroscope will be modified under the consideration of various manufacturing errors. Finally, in order to better design the microgyroscope control system and realize the control objective, the non dimensional processing and the equivalent transformation of the model are carried out. The mechanical structure of the micro gyroscope can be understood as a proof mass attached to a rigid frame by springs and dampers, as shown in Fig 1.

For the z-axis gyroscope, the basic mass block is limited in the X-Y plane, the main vibration direction is the direction of the drive along the X axis and the direction of induction along the Y axis. Dynamic equations of the microgyroscope can be obtained by Newton's law in rotating frame. Considering the influence of various manufacturing defects of the microgyroscope and linearizing the dynamical model, the vibration equation of the microgyroscope is modified as:

$$\begin{array}{l} m\ddot{x}+d_{xx}\dot{x}+d_{xy}\dot{y}+k_{xx}x+k_{xy}y=u_{x}+2m\Omega_{z}\dot{y}\\ m\ddot{y}+d_{xy}\dot{x}+d_{yy}\dot{y}+k_{xy}x+k_{yy}y=u_{y}-2m\Omega_{z}\dot{x} \end{array}$$ (1)

where *m* is the mass of mass block, *d*_*xx*_ and *d*_*yy*_ are the damping coefficients of x-axis and y-axis, respectively, *k*_*xx*_ and *k*_*yy*_ are the spring coefficients of x-axis and y-axis, respectively, *k*_*xy*_ and *d*_*xy*_ are the coupling coefficient and damping coefficient caused by manufacturing error, *u*_*x*_,*u*_*y*_ are the control inputs of x-axis and y-axis, *x* and *y* are the coordinates of x-axis and y-axis in the rotating coordinate system, Ω_*z*_ is angular velocity in the z direction.

Eq (1) is a dimensional mathematical model of microgyroscope, in other words, the physical quantity in the equation should not only consider the numerical value, but also consider the consistency of the physical unit, therefore, the complexity of controller design is increased. In order to solve the aforementioned problem, it is necessary to carry out the dimensionless processing for the mathematical model of microgyroscope.

Both sides of the Eq (1) are divided by $m,q_{0},\omega_{0}^{2}$, then the non-dimensional form can be obtained. *m* is the mass of mass block, *q*_0_ is the reference length, $\omega_{0}^{2}$ is the square of the resonance frequency of the two axis. So the dimensionless model is obtained as follows:

$$\begin{array}{l} \ddot{x}+d_{xx}\dot{x}+d_{xy}\dot{y}+\omega_{x}{}^{2}x+\omega_{xy}y=u_{x}+2\Omega_{z}\dot{y}\\ \ddot{y}+d_{xy}\dot{x}+d_{yy}\dot{y}+\omega_{xy}x+\omega_{y}{}^{2}y=u_{y}-2\Omega_{z}\dot{x} \end{array}$$ (2)

where $\frac{d_{xx}}{m\omega_{0}}\to d_{xx},\;\frac{d_{xy}}{m\omega_{0}}\to d_{xy},\;\frac{d_{yy}}{m\omega_{0}}\to d_{yy},\frac{k_{xx}}{m\omega_{0}{}^{2}}\to \omega_{x}^{2},\;\frac{k_{xy}}{m\omega_{0}{}^{2}}\to \omega_{xy}$ $\frac{k_{yy}}{m\omega_{0}{}^{2}}\to \omega_{y}^{2},\frac{\Omega_{z}}{m\omega_{0}}\to \Omega_{z}$.

Therefore, the model described in Eq (2) can be rewritten into vector form as:

$$\ddot{q}+D\dot{q}+Kq=u-2\Omega \dot{q}$$ (3)

where $q=\left[\begin{array}{c} x\\ y \end{array}\right],D=\left[\begin{array}{cc} d_{xx} & d_{xy}\\ d_{xy} & d_{yy} \end{array}\right],K=\left[\begin{array}{cc} \omega_{x}^{2} & \omega_{xy}\\ \omega_{xy} & \omega_{y}^{2} \end{array}\right],u=\left[\begin{array}{c} u_{x}\\ u_{y} \end{array}\right],\Omega =\left[\begin{array}{cc} 0 & -\Omega_{z}\\ \Omega_{z} & 0 \end{array}\right]$.

Considering the parameter uncertainties and external disturbances of the system, according to the equivalent model of the microgyroscope system described in Eq (3), the model of the microgyroscope system can be modified as:

$$\ddot{q}+(D+2\Omega +\Delta D)\dot{q}+(K+\Delta K)q=u+d$$ (4)

where Δ*D* is the uncertainty of the unknown parameters of the inertia matrix *D* + 2Ω, Δ*K* is the uncertainty of the unknown parameters of matrix *K*. *d* is external disturbance.

Further, the Eq (4) can be expressed as:

$$\ddot{q}+(D+2\Omega )\dot{q}+Kq=u+\varphi (t)$$ (5)

***Remark 1***: *φ*(*t*) is the uncertainty of the lumped parameter and disturbance of the system, the derivative of uncertainty and external disturbance satisfy $\left|\dot{\varphi }(t)\right|\leq \delta$, where *δ* is the upper bound of the derivative of the uncertainty and disturbance, which is a positive constant.

### Super-Twisting sliding mode control

The control signal of the sliding mode control usually can be divided into two parts, one regarding the equivalent control, which deals with the dynamics of the system and the sliding surface, and another regarding the switching control, which is responsible for keeping the dynamics of the system onto the sliding surface.

Defining the sliding surface as:

$$s=ce+\dot{e}$$ (6)

where *c* is a sliding coefficient, *e* and $\dot{e}$ are the tracking error and the derivative of tracking error, respectively. They are defined as follows:

$$e=q-q_{r}={\left[q_{1}-q_{r1},q_{2}-q_{r2}\right]}^{T}$$ (7)

$$\dot{e}=\dot{q}-{\dot{q}}_{r}={\left[{\dot{q}}_{1}-{\dot{q}}_{r1},{\dot{q}}_{2}-{\dot{q}}_{r2}\right]}^{T}$$ (8)

where *q*_*r*_ is the desired trajectory and *q* is the actual trajectory.

The sliding mode control is given by:

$$u=u_{eq}+u_{sw}$$ (9)

where *u*_*eq*_ is the equivalent control proposed by Filipov without considering the system uncertainty and external disturbance. It serves to hold the variable to control on the sliding surfaces. The equivalent control is derived by considering that the derivative of the surface is null $\dot{s}=0$. *u*_*sw*_ is the discrete control, which ensures convergence such that: $s\dot{s}<0$.

In traditional sliding mode control, switching control is usually adopted as:

$$u_{sw}=-k\mathrm{sign}(s)$$ (10)

where *k* is a positive constant, *sign*(*s*) is symbolic function defined by:

$$sign(s)=\left\{ \begin{array}{lll} -1 & \;if & \;s<0\\ 0 & \;if & \;s=0\\ +1 & \;if & \;s>0 \end{array}\right.$$ (11)

But there is a problem with this switching control, which will lead to serious chattering in high frequency switch, degrading the performance of the conventional sliding mode control.

High order sliding mode control can not only provide the same advantages as traditional advantages in terms of robustness, but also can eliminate or weaken the chattering, while providing high precision. Super-Twisting sliding mode control is one of the higher order sliding mode control, which can avoid the chattering while maintaining the other sliding modes properties, containing two parts, one is a discontinuous function of the sliding variable, and the other is a continuous function of its derivative. which can be expressed

$$u_{sw}=-k_{1}\sqrt{\left|s\right|}\mathrm{sgn}(s)+v$$ (12)

$$\dot{v}=-k_{2}\mathrm{sgn}(s)$$ (13)

where *k*_1_ and *k*_2_ are positive constants.

### Adaptive Super-Twisting sliding mode control system

**Fig 2:**
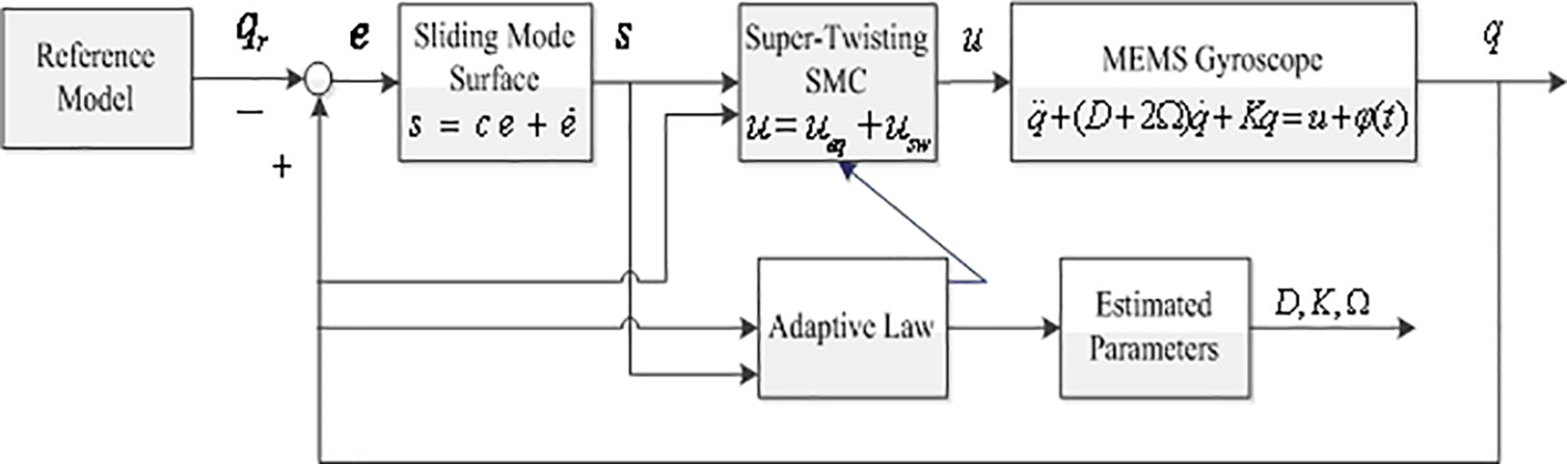
Block diagram of adaptive Super-Twisting sliding mode control for microgyroscope.

The aim of the control is to design a suitable control law, so that the output of the system can track the reference trajectory quickly and precisely in a finite time. In order to solve the trajectory tracking problem of microgyroscope with unknown model uncertainties and external disturbances, a new adaptive Super-Twisting sliding mode control method is proposed in this section. Adaptive control provides an effective method to solve the problem of uncertain system, especially the unknown parameters of the microgyroscope system can be solved according to the adaptive control method. Super-Twisting sliding mode control can achieve the robustness of the uncertainties and external disturbances, and effectively restrain the chattering. This method has strong robustness, fast convergence speed and high precision. The block diagram of adaptive Super-Twisting sliding mode control for microgyroscope is shown in Fig 2.

#### Design of controller

The control law is designed by combining the equivalent control and the Super-Twisting control algorithm, first, select the following control algorithm:

$$u(t)=u_{con}+u_{dis}$$ (14)

where *u*_*con*_ is a continuous control part, which can be regarded as the equivalent control *u*_*eq*_ without considering the system uncertainty and external disturbance disturbance, to guarantee the state of the system on the sliding surface. *u*_*dis*_ is a discontinuous control part, which can be regarded as the switching control *u*_*sw*_ to realize the robust control of external disturbance and uncertainty and weaken the chattering. Especially the switching control is designed by the Super-Twisting control algorithm as in (12),(13).

Therefore, the time derivative of sliding surface *s* is:

$$\dot{s}=c\dot{e}+\ddot{e}=c\dot{e}+\ddot{q}-{\ddot{q}}_{r}$$ (15)

Without considering the system uncertainty and external disturbance, Eq (5) can be written as:

$$\ddot{q}+(D+2\Omega )\dot{q}+Kq=u$$ (16)

Substituting Eq (16) into Eq (15) generates:

$$\dot{s}=c\dot{e}-(D+2\Omega )\dot{q}-Kq+u-{\ddot{q}}_{r}$$ (17)

Setting $\dot{s}=0$, then the equivalent control law can be obtained as:

$$u_{eq}=-c\dot{e}+(D+2\Omega )\dot{q}+Kq+{\ddot{q}}_{r}$$ (18)

The switching control law is designed based on the Super-Twisting algorithm, the algorithm is as follows:

$$u_{sw}=-k_{1}\sqrt{\left|s\right|}\mathrm{sgn}(s)-\int k_{2}\mathrm{sgn}(s)dt$$ (19)

where *k*_1_ and *k*_2_ are positive constants.

***Remark 2*:** In order to ensure the stability of the microgyroscope the value of *k*_2_ must satisfy $k_{2}>\delta >\left|\dot{\varphi }(t)\right|$.

The final control law can be obtained as follows:

$$u=-c\dot{e}+(D+2\Omega )\dot{q}+Kq+{\ddot{q}}_{r}-k_{1}\sqrt{\left|s\right|}\mathrm{sgn}(s)-\int k_{2}\mathrm{sgn}(s)dt$$ (20)

***Remark 3*:** Super-Twisting sliding mode control can make the system states reach the equilibrium point in a finite time, the brief mathematical expression of convergence time can be written as:

$$\begin{array}{l} T=\frac{2}{\gamma \left(Q\right)}V^{\frac{1}{2}}(x(0),y(0))=\frac{2\lambda_{\max}^{\frac{1}{2}}(P)}{\lambda_{\min}(Q)}V^{\frac{1}{2}}(x(0),y(0))\\ \;\leq \frac{2\lambda_{\max}^{\frac{1}{2}}(P)\lambda_{\max}^{\frac{1}{2}}(P)}{\lambda_{\min}(Q)}{\left\|\varsigma (0)\right\|}_{2}\\ \;=\frac{2\lambda_{\max}^{}(P)}{\lambda_{\min}(Q)}{\left\|\varsigma (0)\right\|}_{2}\\ \;=2\beta (P,Q){\left\|\varsigma (0)\right\|}_{2} \end{array}$$ (21)

where $\beta (P,Q)=\frac{\lambda_{\max}^{}(P)}{\lambda_{\min}(Q)}$, $\gamma (Q)=\frac{\lambda_{\min}(Q)}{\lambda_{\max}^{\frac{1}{2}}(P)}$, $P=\frac{1}{2}\left[\begin{array}{cc} 4k_{2}+k_{1}{}^{2} & -k_{1}\\ -k_{1} & 2 \end{array}\right]$ $\varsigma^{T}=\left[\varsigma_{1},\varsigma_{2}\right]=\left[{\left|s\right|}^{\frac{1}{2}}\mathrm{sgn}(s),y\right]$, *V*(*x*,y) = *ς*^*T*^**P***ς*,

$$Q=\left[\begin{array}{cc} k_{1}k_{2}+\frac{k_{1}{}^{3}}{2}-\delta^{2}-\frac{k_{1}{}^{2}}{4} & \frac{k_{1}}{2}-\frac{k_{1}{}^{2}}{2}\\ \frac{k_{1}}{2}-\frac{k_{1}{}^{2}}{2} & \frac{k_{1}}{2}-1 \end{array}\right],\left\{ \begin{array}{l} \dot{s}=-k_{1}\sqrt{\left|s\right|}\mathrm{sgn}(s)+y\\ \dot{y}=-k_{2}\mathrm{sgn}(s)+\dot{\varphi }(t) \end{array}\right.$$

The estimation of the convergence time T is optimal when **Q** = **I**, and **I** is the unit matrix.

#### Design of adaptive law and stability analysis

For the actual microgyroscope, three parameters *D*,*K*,Ω are unknown or can not be obtained accurately, therefore, the control law of Eq (18) can not be implemented directly. According to the general idea of adaptive control, using estimated values $\hat{D},\hat{K},\hat{\Omega }$ to replace the unknown true values *D*,*K*,Ω and designing adaptive algorithms of the three parameters *D*,*K*,Ω to estimate and update the unknown parameters and angular velocity of microgyroscope.

Consequently, Eq (18) can be rewritten as:

$$u_{eq'}=-c\dot{e}+(\hat{D}+2\hat{\Omega })\dot{q}+\hat{K}q+{\ddot{q}}_{r}$$ (22)

Accordingly, Eq (20) becomes:

$$u=-c\dot{e}+(\hat{D}+2\hat{\Omega })\dot{q}+\hat{K}q+{\ddot{q}}_{r}-k_{1}\sqrt{\left|s\right|}\mathrm{sgn}(s)-\int k_{2}\mathrm{sgn}(s)dt$$ (23)

According to Lyapunov stability theory to design the adaptive algorithms of $\hat{D},\hat{K},\hat{\Omega }$, the estimation errors of *D*,*K*,Ω is defined as:

$$\left\{ \begin{array}{l} \tilde{D}=\hat{D}-D\\ \tilde{K}=\hat{K}-K\\ \tilde{\Omega }=\hat{\Omega }-\Omega \end{array}\right.$$ (24)

Substituting Eq (23) into Eq (10) generates:

$$\ddot{q}-{\ddot{q}}_{r}+c\dot{e}=-(D+2\Omega )\dot{q}+(\hat{D}+2\hat{\Omega })\dot{q}-Kq+\hat{K}q-k_{1}\sqrt{\left|s\right|}\mathrm{sgn}(s)-\int k_{2}\mathrm{sgn}(s)dt+\varphi (t)$$ (25)

Substituting Eq (15) into Eq (25) generates:

$$\dot{s}=-(D+2\Omega )\dot{q}+(\hat{D}+2\hat{\Omega })\dot{q}-Kq+\hat{K}q-k_{1}\sqrt{\left|s\right|}\mathrm{sgn}(s)-\int k_{2}\mathrm{sgn}(s)dt+\varphi (t)$$ (26)

According to the definition of parameter estimation errors based on Eq (24), further, the Eq (24) can be simplified as:

$$\dot{s}=(\tilde{D}+2\tilde{\Omega })\dot{q}+\tilde{K}q+u_{sw}+\varphi (t)$$ (27)

***Theorem 1*.** If the control law (23), the parameter adaptive laws of $\hat{D},\hat{K},\hat{\Omega }$ designed as Eq (28) are adopted in the microgyroscope represented by Eq (5), then the output tracking error e(t) will converge to zero asymptotically and all the unknown gyroscope parameters including the angular rate can be estimated correctly.

$$\left\{ \begin{array}{l} \dot{\hat{D}}=-\frac{1}{2}(s{\dot{q}}^{T}+\dot{q}s^{T})M^{T}\\ \dot{\hat{K}}=-\frac{1}{2}(sq^{T}+qs^{T})N^{T}\\ \dot{\hat{\Omega }}=-(s{\dot{q}}^{T}-\dot{q}s^{T})P^{T} \end{array}\right.$$ (28)

where *M* = *M*^*T*^ > 0,*N* = *N*^*T*^ > 0,*P* = *P*^*T*^ > 0, they are positive definite symmetric matrices.

***Proof*:** The Lyapunov function candidate is defined as:

$$V=\frac{1}{2}s^{T}s+\frac{1}{2}tr\left\{\tilde{D}M^{-1}{\tilde{D}}^{T}\right\}+\frac{1}{2}tr\left\{\tilde{K}N^{-1}{\tilde{K}}^{T}\right\}+\frac{1}{2}tr\left\{\tilde{\Omega }P^{-1}{\tilde{\Omega }}^{T}\right\}$$ (29)

where *tr*{•} represents the inverse operation of the matrix.

Then the derivative of *V* can be obtained as:

$$\dot{V}=s^{T}\dot{s}+tr\left\{\tilde{D}M^{-1}{\dot{\tilde{D}}}^{T}\right\}+tr\left\{\tilde{K}N^{-1}{\dot{\tilde{K}}}^{T}\right\}+tr\left\{\tilde{\Omega }M^{-1}{\dot{\tilde{\Omega }}}^{T}\right\}$$ (30)

Substituting Eq (27) into Eq (30) generates:

$$\begin{array}{l} \dot{V}=s^{T}(\tilde{D}\dot{q}+2\tilde{\Omega }\dot{q}+\tilde{K}q+u_{sw}+\varphi (t))+tr\left\{\tilde{D}M^{-1}{\dot{\tilde{D}}}^{T}\right\}+tr\left\{\tilde{K}N^{-1}{\dot{\tilde{K}}}^{T}\right\}+tr\left\{\tilde{\Omega }M^{-1}{\dot{\tilde{\Omega }}}^{T}\right\}\\ \;=s^{T}(u_{sw}+\varphi (t))+s^{T}\tilde{D}\dot{q}+tr\left\{\tilde{D}M^{-1}{\dot{\tilde{D}}}^{T}\right\}+s^{T}\tilde{K}q+tr\left\{\tilde{K}N^{-1}{\dot{\tilde{K}}}^{T}\right\}+2s^{T}\tilde{\Omega }\dot{q}+tr\left\{\tilde{\Omega }M^{-1}{\dot{\tilde{\Omega }}}^{T}\right\} \end{array}$$ (31)

Because *D* = *D*^*T*^,*K* = *K*^*T*^,Ω = −Ω^*T*^, $s^{T}\tilde{D}\dot{q}={\dot{q}}^{T}\tilde{D}s$ (It is scalar), the following equation will be obtained:

$$s^{T}\tilde{D}\dot{q}=\frac{1}{2}(s^{T}\tilde{D}\dot{q}+{\dot{q}}^{T}\tilde{D}s)$$ (32)

Similarly:

$$s^{T}\tilde{K}q=\frac{1}{2}(s^{T}\tilde{K}q+q^{T}\tilde{K}s)$$ (33)

$$2s^{T}\tilde{\Omega }\dot{q}=\frac{1}{2}(2s^{T}\tilde{\Omega }\dot{q}-2{\dot{q}}^{T}\tilde{\Omega }s)$$ (34)

Therefore, Eq (29) can be modified as:

$$\begin{array}{l} \dot{V}=s^{T}(u_{sw}+\varphi (t))+tr\left\{\tilde{D}\left[M^{-1}{\dot{\hat{D}}}^{T}+\frac{1}{2}(\dot{q}s^{T}+s{\dot{q}}^{T})\right]\right\}\\ \;+tr\left\{\tilde{K}\left[N^{-1}{\dot{\hat{K}}}^{T}+\frac{1}{2}(qs^{T}+sq^{T})\right]\right\}+tr\left\{\tilde{\Omega }\left[P^{-1}{\dot{\hat{\Omega }}}^{T}+\frac{1}{2}(2\dot{q}s^{T}-2s{\dot{q}}^{T})\right]\right\} \end{array}$$ (35)

In order to ensure $\dot{V}\leq 0$, substituting adaptive laws of Eq (28) into Eq (35) generates:

$$\begin{array}{l} \dot{V}=s^{T}(u_{sw}+\varphi (t))\\ \;=s^{T}(-k_{1}\sqrt{\left|s\right|}\mathrm{sgn}(s)-\int k_{2}\mathrm{sgn}(s)dt+\varphi (t))\\ \;=-s^{T}k_{1}\sqrt{\left|s\right|}\mathrm{sgn}(s)-s^{T}\int k_{2}\mathrm{sgn}(s)dt+s^{T}\varphi (t)\\ \;\leq -k_{1}\left|s^{T}\right|\sqrt{\left|s\right|}-\left|s^{T}\right|\int k_{2}dt+\left|s^{T}\varphi (t)\right|\\ \;=-k_{1}\left|s^{T}\right|\sqrt{\left|s\right|}-\left|s^{T}\right|\int k_{2}dt+\left|s^{T}\right|\left|\varphi (t)\right|\\ \;=-k_{1}\left|s^{T}\right|\sqrt{\left|s\right|}-\left|s^{T}\right|\int k_{2}dt+\left|s^{T}\right|\left|\int \dot{\varphi }(t)dt\right|\\ \;=-k_{1}\left|s^{T}\right|\sqrt{\left|s\right|}-\left|s^{T}\right|\int k_{2}dt+\left|s^{T}\right|\int \left|\dot{\varphi }(t)\right|dt \end{array}$$ (36)

Since $\left|\dot{\varphi }(t)\right|<\delta <k_{2}$, Eq (34) can be rewritten as:

$$\begin{array}{l} \dot{V}\leq -k_{1}\left|s^{T}\right|\sqrt{\left|s\right|}-\left|s^{T}\right|\int k_{2}dt+\left|s^{T}\right|\int \delta dt\\ \;=-k_{1}\left|s^{T}\right|\sqrt{\left|s\right|}-\left|s^{T}\right|(\int k_{2}dt-\int \delta dt)\\ \;\leq -k_{1}\left|s^{T}\right|\sqrt{\left|s\right|}\\ \;\leq 0 \end{array}$$ (37)

Since $\dot{V}\leq 0$, $\dot{V}$ is negative semi-definite. Hence the global asymptotic stability of the system can be guaranteed, which also implies the tracking error is uniformly ultimate bounded and all the variables are bounded, such as $\tilde{D},\tilde{K},\tilde{\Omega }$ are all bounded. According to Barbalat lemma, *s*(*t*) will asymptotically converge to zero, lim_*t*→∞_ *s*(*t*) = 0, that is, *s*(*t*) and *e*(*t*) all converge to zero asymptotically. In addition, the derivative of sliding surface $\dot{s}$ can asymptotically converge to zero in a finite time. This indicates that the sliding mode condition is satisfied and the robustness of stability can be guaranteed.

## Simulation study

In this section, in order to demonstrate the effectiveness of the proposed adaptive Super-Twisting sliding mode control scheme, simulation studies were implemented in Matlab/Simulink environment for both adaptive Super-Twisting sliding mode control and conventional adaptive sliding mode control approaches. Parameters of adopted microgyroscope are as follows:

$$\begin{array}{l} m=1.8\times 10^{-7}\mathrm{kg},\;k_{xx}=63.955N/m,k_{yy}=95.92N/m,\;k_{xy}=12.779N/m\\ d_{xx}=1.8\times 10^{-6}N\;s/m,\;d_{yy}=1.8\times 10^{-6}N\;s/m,d_{xy}=3.6\times 10^{-7}N\;s/m \end{array}$$

The angular velocity of the input is assumed to be Ω_*z*_ = 100rad/s, the reference length is *q*_0_ = 1μm, the reference frequency is *ω*_0_ = 1000*Hz*. The dimensionless parameters of the microgyroscope are listed after dimensionless processing:

$$\begin{array}{l} \omega_{x}{}^{2}=355.3,\;\omega_{y}{}^{2}=532.9,\;\omega_{xy}=70.99,\;d_{xx}=0.01\\ d_{yy}=0.01,\;d_{xy}=0.002,\;\Omega_{z}=0.1 \end{array}$$

In the simulation, the initial condition of the system is selected as $q_{1}(0)=1.0,{\dot{q}}_{1}(0)=0,q_{2}(0)=0.5,{\dot{q}}_{2}(0)=0$. The desired trajectory of the two axis of the microgyroscope is *q*_*r*1_ = sin(*πt*),*q*_*r*2_ = cos(0.5*πt*). The estimated value of the three parameter matrix is $\hat{D}(0)=0.95*D,\hat{K}(0)=0.95*K,\hat{\Omega }(0)=0$, the adaptive gain of Eq (35) is *M* = *N* = *P* = *diag*(150,150). The sliding coefficient is selected as *c* = 10. As for model uncertainties, we allow ±30% parameter variations for the spring and damping coefficients with respect to their nominal values and ±30% magnitude changes in the coupling terms, that is, *d*_*xy*_ and *ω*_*xy*_. Random signal *d* = [0.5 * *randn*(1,1);0.5*randn(1,1)] is considered as external disturbance. When we adopt adaptive Super-Twisting sliding mode control, the Super-Twisting sliding mode controller parameters of Eq (22) are selected as *k*_1_ = 15, k_2_ = 5. However when the conventional adaptive sliding mode control is simulated, the sliding controller parameter of Eq (6) is selected as *k* = 6.

In the simulation, the simulation time is set as 60s, and the simulation results are shown in Figs 3–14.

**Fig 3:**
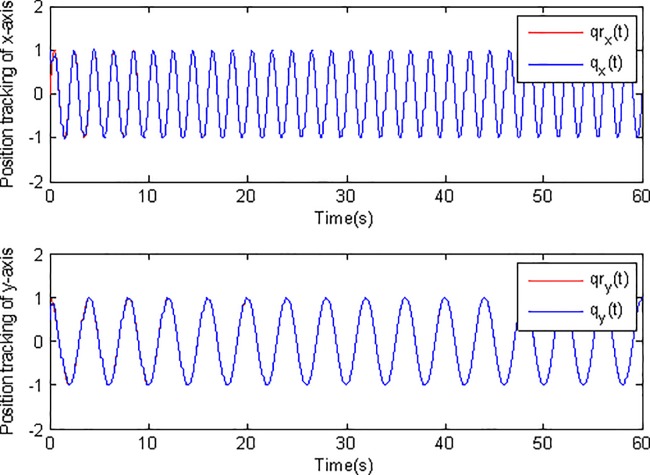
Position tracking using adaptive Super-Twisting sliding mode control.

**Fig 4:**
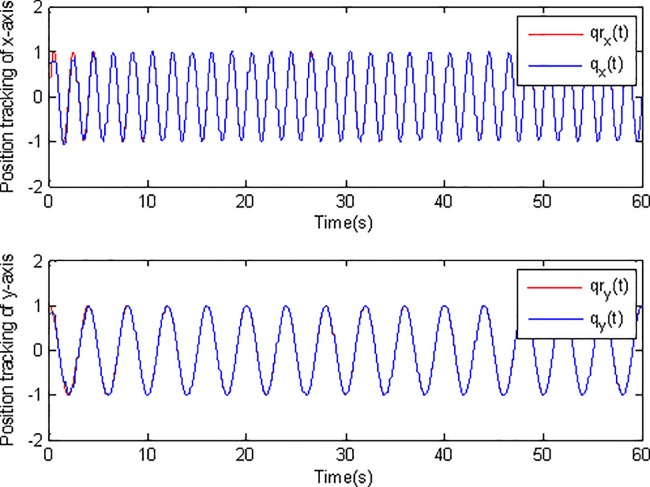
Position tracking using conventional adaptive sliding mode control.

**Fig 5:**
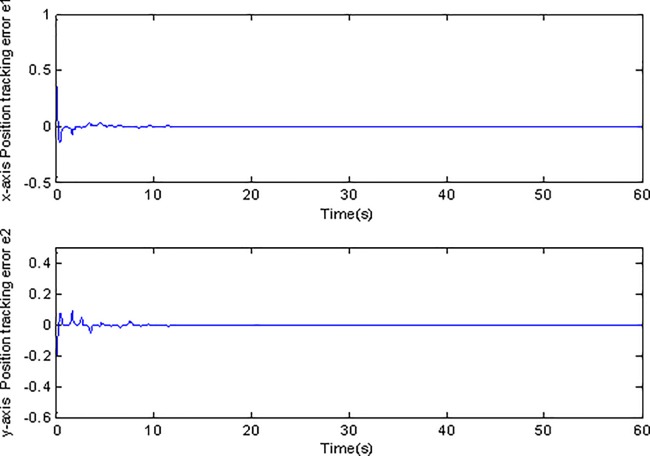
Tracking errors using adaptive Super-Twisting sliding mode control.

**Fig 6:**
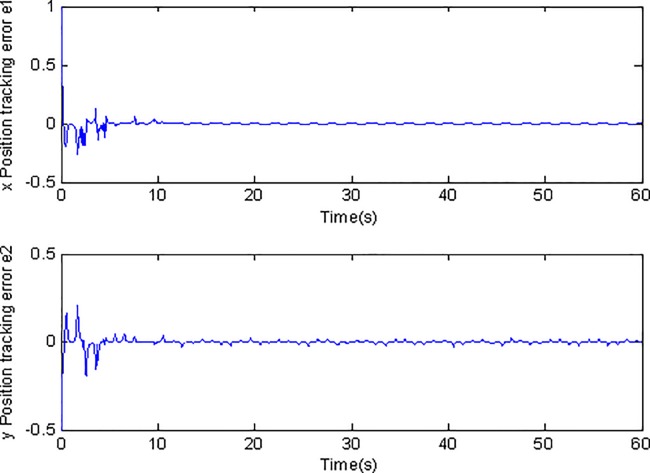
Tracking errors using conventional adaptive sliding mode control.

**Fig 7:**
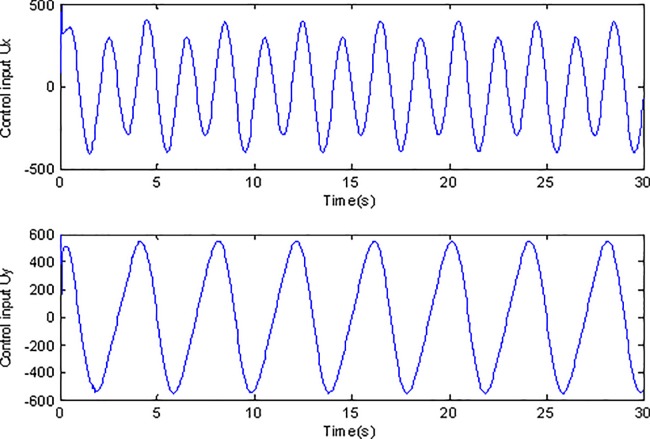
Control inputs using adaptive Super-Twisting sliding mode control.

**Fig 8:**
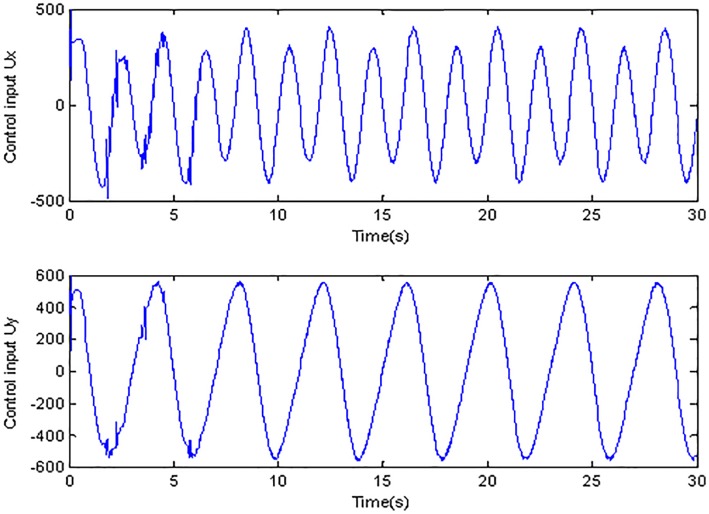
Control inputs using conventional adaptive sliding mode control.

**Fig 9:**
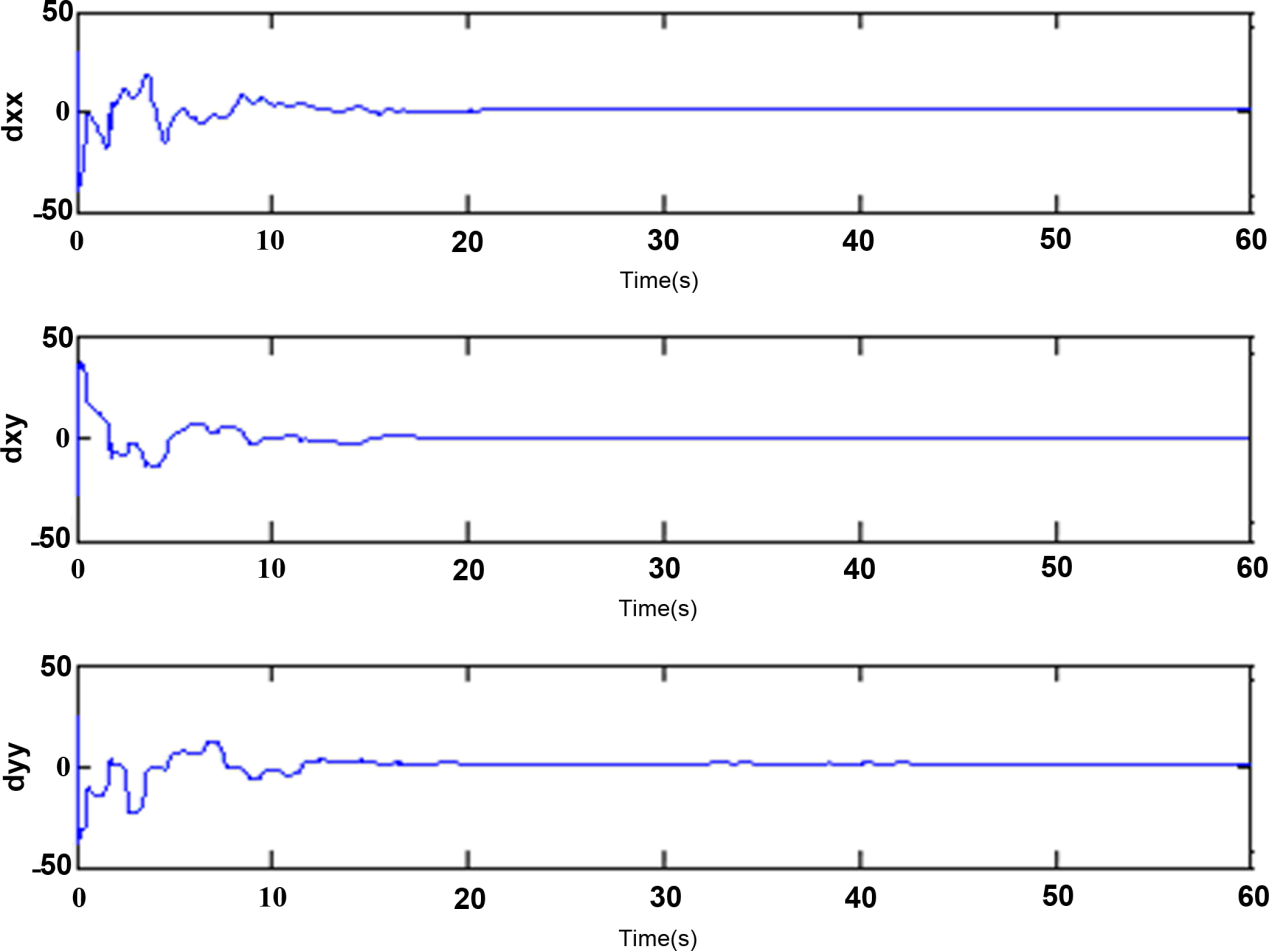
Estimated values of *d*_*xx*_,*d*_*xy*_,*d*_*yy*_ using adaptive Super-Twisting sliding mode control.

**Fig 10:**
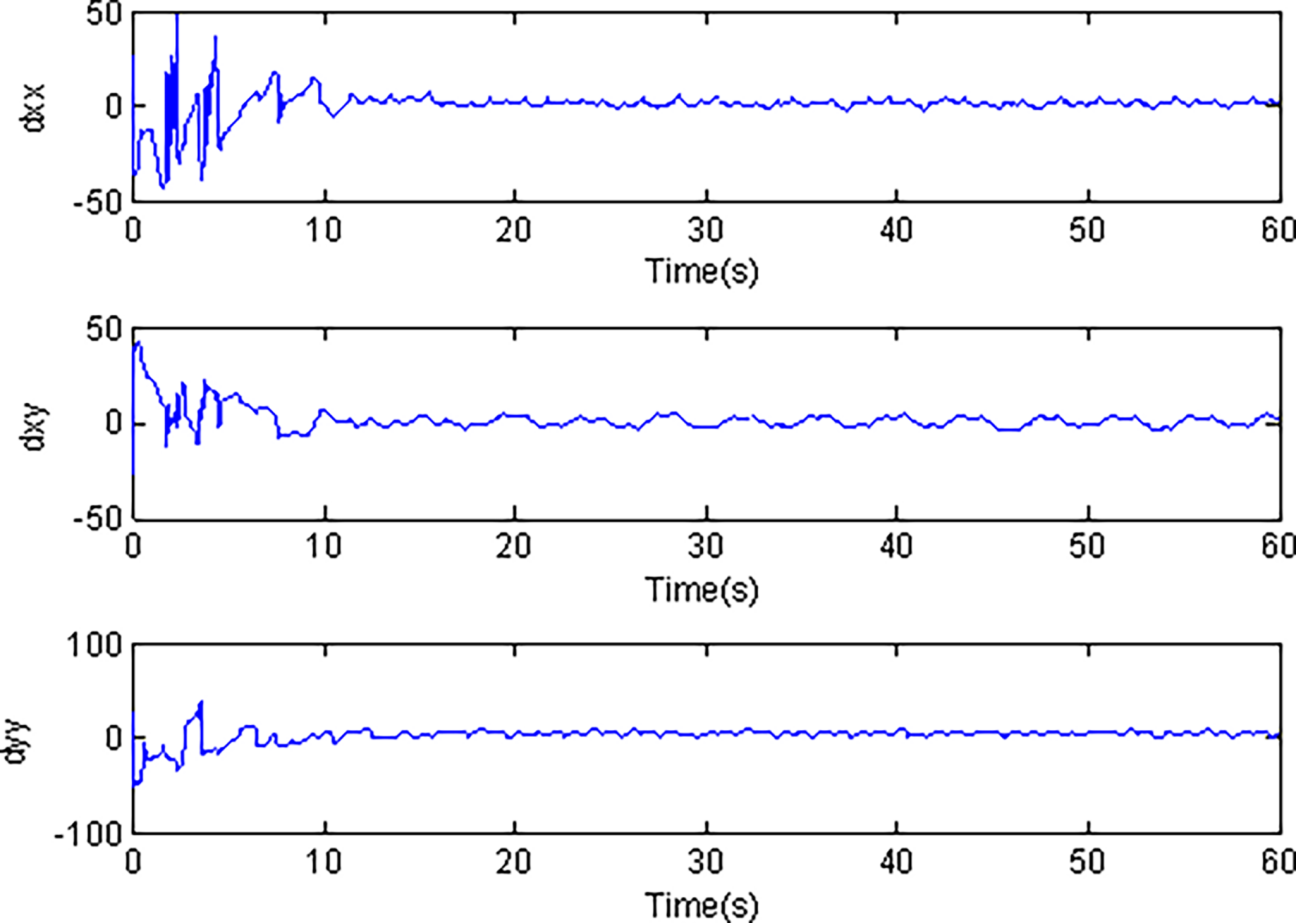
Estimated values of *d*_*xx*_,*d*_*xy*_,*d*_*yy*_ using conventional adaptive sliding mode control.

**Fig 11:**
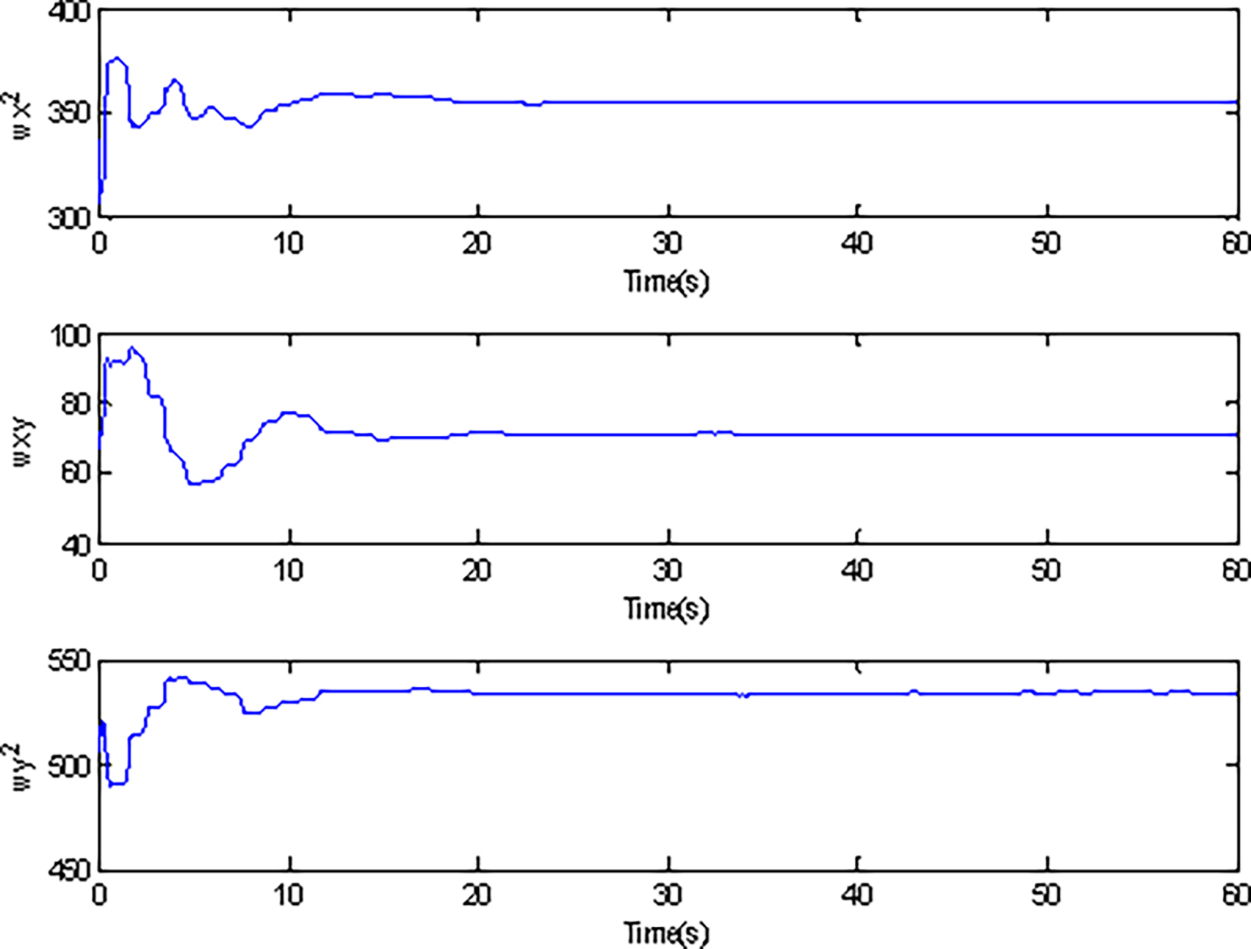
Estimated values of $\omega_{x}^{2},\omega_{xy},\omega_{y}^{2}$ using adaptive Super-Twisting sliding mode control.

**Fig 12:**
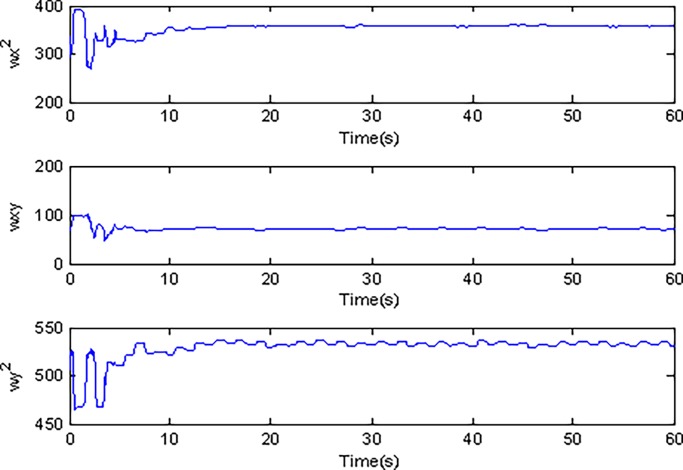
Estimated values of $\omega_{x}^{2},\omega_{xy},\omega_{y}^{2}$ using conventional adaptive sliding mode control.

**Fig 13:**
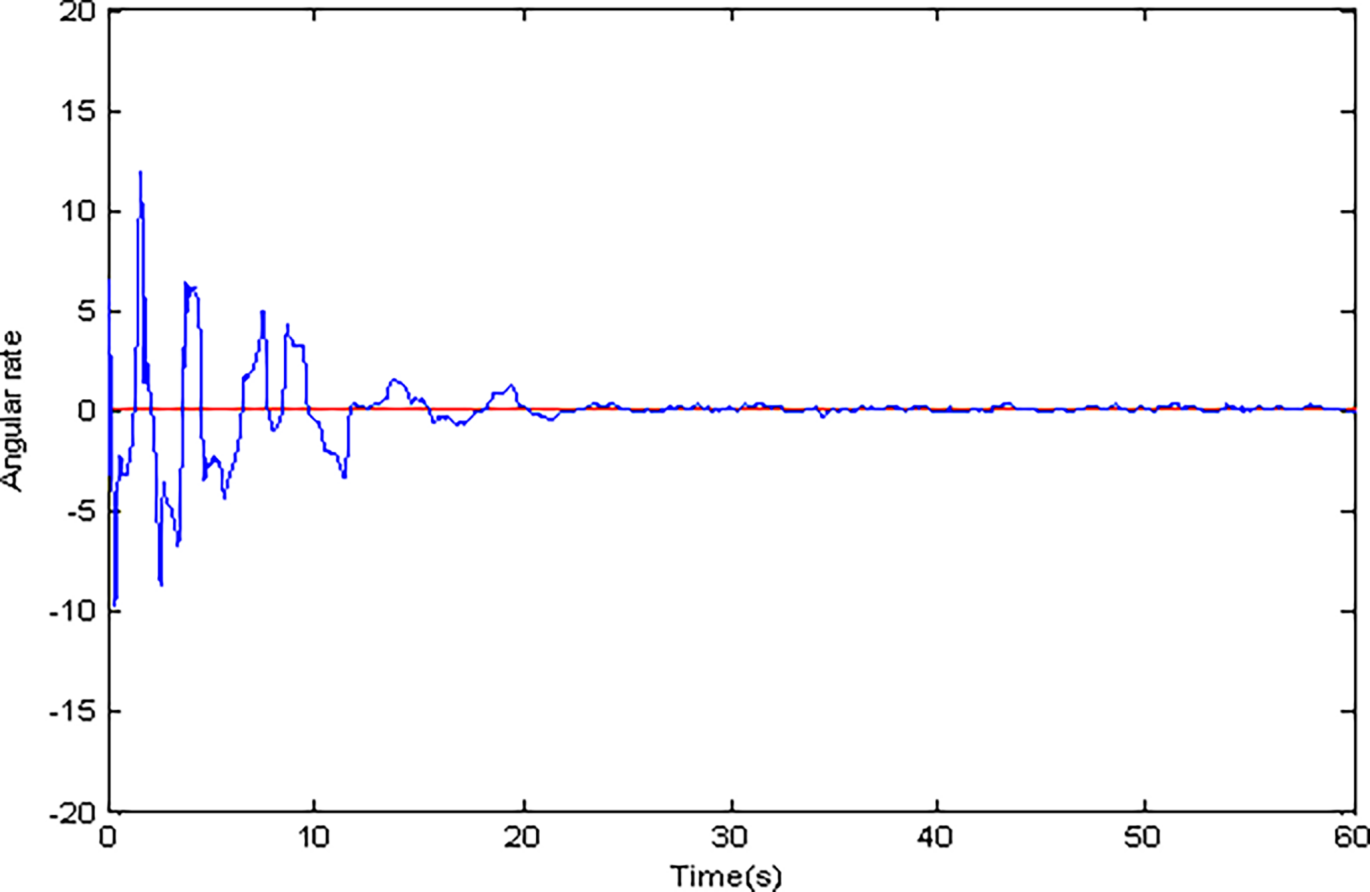
Estimated value of Ω_*z*_ using adaptive Super-Twisting sliding mode control.

**Fig 14:**
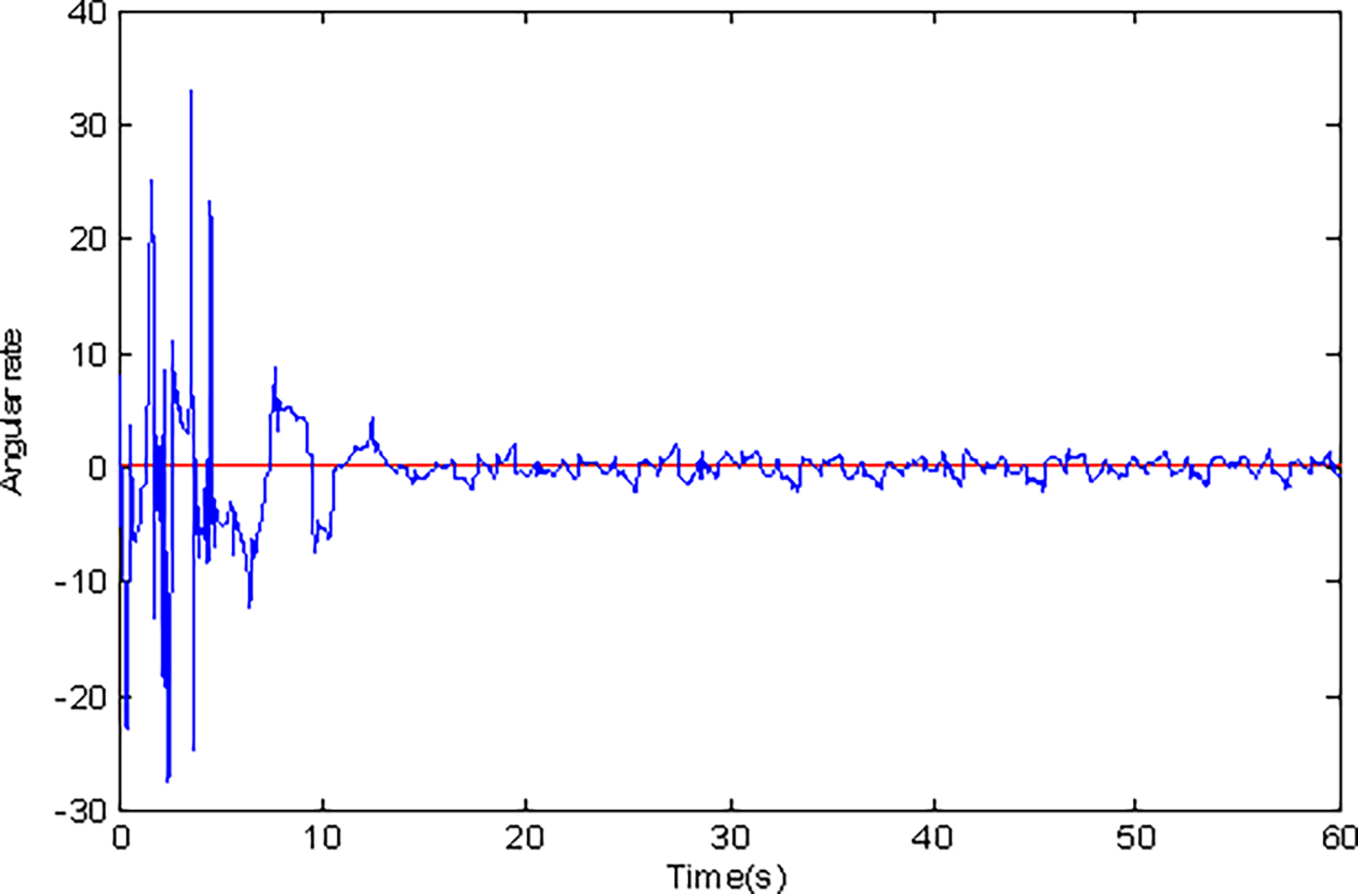
Estimated value of Ω_*z*_ using conventional adaptive sliding mode control.

Fig 3 and Fig 4 show the position tracking under adaptive Super-Twisting sliding mode control and conventional adaptive sliding mode control respectively. Fig 5 and Fig 6 draw the tracking errors under the two cases respectively. It can be seen that the proposed adaptive Super-Twisting sliding mode controller can achieve more accurate and effective tracking and can reach the desired reference trajectory effectively in a shorter finite time than the method in Fig 4. Fig 5 and Fig 6 show that both two controllers can make the tracking errors decrease and converge to zero quickly. However, the proposed adaptive Super-Twisting sliding mode controller has a faster reduction rate in tracking errors than the method based on conventional adaptive sliding mode control. In summary, adaptive Super-Twisting sliding mode control has a better tracking property than conventional adaptive sliding mode control.

Fig 7 and Fig 8 describe the control inputs under two cases. Due to the high frequency of the selected reference trajectory, the simulation time is set to 30s in order to show the superiority of the control method in this paper. Compared with Fig 8, it is obvious that the chattering is restrained effectively under adaptive Super-Twisting sliding mode control while the chattering phenomenon is obvious in Fig 8. Because the traditional sliding mode control utilizes the sign function *sign*(*s*), when the s swings around 0, the control input will change at high frequency, after multiplying the correlation coefficient, the amplitude is obviously enlarged. However the method proposed in this paper utilizes the Super-Twisting algorithm, which can reduce the chattering effectively.

Adaptive identification curve of microgyroscope are shown in Fig 9 to Fig 12. It is observed that the estimated values of *d*_*xx*_,*d*_*xy*_,*d*_*yy*_ and $w_{x}^{2},w_{xy},w_{y}^{2}$ under the adaptive Super-Twisting sliding mode control can converge to their true values in shorter time and have smaller overshoot than that under the conventional adaptive sliding mode control.

Fig 13 and Fig 14 indicate the estimated value of Ω_*z*_ under two cases. It is obvious that the adaptive Super-Twisting sliding mode control has a better estimation effect. Simulation results also verify that the estimated value of Ω_*z*_ under the adaptive Super-Twisting sliding mode control can converge to its true value in shorter time and overshoot is smaller than that under the conventional adaptive sliding mode control.

All these simulation results prove the superiority and validity of the proposed method in this paper. This method is superior to the traditional adaptive sliding mode control in all aspects, Therefore, we can obtain the satisfactory performance by the proposed adaptive Super-Twisting sliding mode control.

## Conclusion

In this paper, an adaptive Super-Twisting sliding mode control method is proposed, which mainly aimes at the trajectory tracking and the estimation of unknown parameters and the angular velocity of microgyroscope. This method combines the advantages of high order Super-Twisting sliding mode control and the adaptive control. It can not only ensure the convergence of the system in a finite time, but also achieve a stable state, and the unknown parameters can be updated online according to the adaptive identification method. Compared with the conventional adaptive sliding mode control, the superiority and effectiveness of the adaptive Super-Twisting sliding mode control have been proved. The results demonstrate that the proposed method can not only make the output of the system track the reference trajectory quickly and precisely, but also can effectively restrain the chattering and make the control input smoother.

## Supporting information

S1 File (PDF)
