# Supplementary material for: Design and analysis of adaptive Super-Twisting sliding mode control for a microgyroscope
Source: PLoS One. 2018 Jan 3;13(1):e0189457. doi: 10.1371/journal.pone.0189457 (PMC5751982; doi:10.1371/journal.pone.0189457)
Supplement: S1 File — (PDF) [file pone.0189457.s001.pdf]

Support information

1. The estimation algorithm of convergence time

Considering the control method of microgyroscope, The final control law can be obtained as follows:

$$u = -c\dot{e} + (D + 2\Omega)\dot{q} + Kq + \ddot{q}_r - k_1\sqrt{|s|}\,\mathrm{sgn}(s) - \int k_2\,\mathrm{sgn}(s)dt \tag{1}$$

the time derivative of sliding surface s is:

$$\dot{s} = c\dot{e} - (D + 2\Omega)\dot{q} - Kq + u + \varphi(t) - \ddot{q}_r \tag{2}$$

Substituting Eq. (1) into Eq. (2) generates:

$$\dot{s} = -k_1\sqrt{|s|}\,\mathrm{sgn}(s) - k_2\int \mathrm{sgn}(s)d\tau + \varphi(t) \tag{3}$$

Transforming Eq.(3) into the following equation:

$$\begin{cases} \dot{s} = -k_1\sqrt{|s|}\,\mathrm{sgn}(s) + \mathrm{y} \\ \dot{y} = -k_2\,\mathrm{sgn}(s) + \dot{\varphi}(t) \end{cases} \tag{4}$$

where $\dot{\varphi}(t) \le \delta$, $\varphi(t)$ is the uncertainty of the lumped parameter and disturbance of the system, δ is the upper bound of the derivative of the uncertainty and disturbance, which is a positive constant and k_1, k_2 satisfy the Eq.(5).

$$\begin{cases} k_1 > 2 \\ k_2 > \dfrac{k_1^3 + \delta^2(4k_1 - 8)}{k_1(4k_1 - 8)} \end{cases} \tag{5}$$

Taking positive definite symmetric matrix as $\mathbf{P} = \dfrac{1}{2}\begin{bmatrix} 4k_2 + k_1^2 & -k_1 \\ -k_1 & 2 \end{bmatrix}$

Defining the Lyapunov function as：

$$V(x, \mathrm{y}) = \varsigma^T \mathbf{P} \varsigma \tag{6}$$

where $\varsigma^T = [\varsigma_1, \varsigma_2] = \left[|s|^{\frac{1}{2}}\mathrm{sgn}(s), y \right]$ and

$$\mathbf{A} = \begin{bmatrix} -\dfrac{1}{2}k_1 & \dfrac{1}{2} \\ -k_2 & 0 \end{bmatrix}, \mathbf{B} = [0,1]^T, \mathbf{C} = [1,0], \tilde{\varphi} = |\varsigma_1|\dot{\varphi} \tag{7}$$

Because $\frac{d|s|}{dt} = \dot{s}\,\mathrm{sgn}(s)$， the derivative of ς is：

$$\dot{\varsigma} = \frac{1}{|\varsigma_1|}(\mathbf{A}\varsigma + \mathbf{B}\tilde{\varphi}) \tag{8}$$

Then the derivative of V can be obtained as：

$$\begin{aligned} \dot{V} &= \frac{1}{|\varsigma_1|}\begin{bmatrix}\varsigma \\ \dot{\varphi}\end{bmatrix}^T \begin{bmatrix}\mathbf{A}^{\mathrm{T}}\mathbf{P}+\mathbf{P}\mathbf{A} & \mathbf{P}\mathbf{B} \\ \mathbf{B}^{\mathrm{T}}\mathbf{P} & \mathbf{0}\end{bmatrix}\begin{bmatrix}\varsigma \\ \dot{\varphi}\end{bmatrix} \\ &\le \frac{1}{|\varsigma_1|}\left\{\begin{bmatrix}\varsigma \\ \dot{\varphi}\end{bmatrix}^T \begin{bmatrix}\mathbf{A}^{\mathrm{T}}\mathbf{P}+\mathbf{P}\mathbf{A} & \mathbf{P}\mathbf{B} \\ \mathbf{B}^{\mathrm{T}}\mathbf{P} & \mathbf{0}\end{bmatrix}\begin{bmatrix}\varsigma \\ \dot{\varphi}\end{bmatrix} + \delta^2\varsigma_1^2 - \dot{\varphi}^2\right\} \\ &\le \frac{1}{|\varsigma_1|}\varsigma^T(\mathbf{A}^{\mathrm{T}}\mathbf{P}+\mathbf{P}\mathbf{A}+\delta^2\mathbf{C}^{\mathrm{T}}\mathbf{C}+\mathbf{P}\mathbf{B}\mathbf{B}^{\mathrm{T}}\mathbf{P})\varsigma \end{aligned} \tag{9}$$

Because $\mathbf{Q} = -(\mathbf{A}^{\mathrm{T}}\mathbf{P}+\mathbf{P}\mathbf{A}+\delta^2\mathbf{C}^{\mathrm{T}}\mathbf{C}+\mathbf{P}\mathbf{B}\mathbf{B}^{\mathrm{T}}\mathbf{P})$， then

$$\dot{V} \le -\frac{1}{|\varsigma_1|}\varsigma^T\mathbf{Q}\varsigma \tag{10}$$

Because $V = \varsigma^T\mathbf{P}\varsigma$ is positive definite quadratic form，there is：

$$\lambda_{\min}(\mathbf{P})\|\varsigma\|_2^2 \le \varsigma^T\mathbf{P}\varsigma \le \lambda_{\max}(\mathbf{P})\|\varsigma\|_2^2 \tag{11}$$

where $\lambda_{\min}(\mathbf{P})$ and $\lambda_{\max}(\mathbf{P})$ represent the minimum eigenvalue and the maximum eigenvalue of the matrix $\mathbf{P}$, respectively, and $\|\bullet\|_2$ represents the 2- norm on the Euclidean space R^2 ,and $\|\varsigma\|_2^2 = \varsigma_1^2 + \varsigma_2^2 = |x| + y^2$, then

$$|\varsigma_1| = |x|^{\frac{1}{2}} \le \|\varsigma\|_2 \le \frac{V^{\frac{1}{2}}}{\lambda_{\min}(\mathbf{P})} \tag{12}$$

So we can get：

$$\begin{aligned} \dot{V} &\le -\frac{1}{|\varsigma_1|}\lambda_{\min}(\mathbf{Q})\|\varsigma\|_2^2 = -\frac{\|\varsigma\|_2}{|\varsigma_1|}\lambda_{\min}(\mathbf{Q})\|\varsigma\|_2 \\ &\le -\lambda_{\min}(\mathbf{Q})\|\varsigma\|_2 \end{aligned} \tag{13}$$

From equation (12), we can get the following equation

$$\frac{V^{\frac{1}{2}}}{\lambda_{\max}^{\frac{1}{2}}(\mathbf{P})} \le \|\varsigma\|_2 \tag{14}$$

Combining equation (13) and equation (14) yields：

$$\dot{V} \le -\gamma(\mathbf{Q})\, V^{\frac{1}{2}}(x, y) \tag{15}$$

where $\gamma(\mathbf{Q}) = \dfrac{\lambda_{\min}(\mathbf{Q})}{\lambda_{\max}^{\frac{1}{2}}(\mathbf{P})}$ ， according to the comparison theorem, $V = 0$ when the t satisfies $t \ge T = \dfrac{2}{\gamma(\mathbf{Q})} V^{\frac{1}{2}}(x_0, y_0)$,therefore, the state of the system represented by (4) can converge to the origin at a finite time T.

then：

$$\mathbf{Q} = \begin{bmatrix} k_1 k_2 + \dfrac{k_1^3}{2} - \delta^2 - \dfrac{k_1^2}{4} & \dfrac{k_1}{2} - \dfrac{k_1^2}{2} \\ \dfrac{k_1}{2} - \dfrac{k_1^2}{2} & \dfrac{k_1}{2} - 1 \end{bmatrix} \tag{16}$$

The convergence time is :

$$\begin{aligned} T &= \frac{2}{\gamma(\mathbf{Q})} V^{\frac{1}{2}}(x(0), y(0)) = \frac{2\lambda_{\max}^{\frac{1}{2}}(\mathbf{P})}{\lambda_{\min}(\mathbf{Q})} V^{\frac{1}{2}}(x(0), y(0)) \\ &\le \frac{2\lambda_{\max}^{\frac{1}{2}}(\mathbf{P})\lambda_{\max}^{\frac{1}{2}}(\mathbf{P})}{\lambda_{\min}(\mathbf{Q})} \|\varsigma(0)\|_2 \\ &= \frac{2\lambda_{\max}(\mathbf{P})}{\lambda_{\min}(\mathbf{Q})} \|\varsigma(0)\|_2 \\ &= 2\beta(\mathbf{P}, \mathbf{Q}) \|\varsigma(0)\|_2 \end{aligned} \tag{17}$$

where $\beta(\mathbf{P}, \mathbf{Q}) = \dfrac{\lambda_{\max}(\mathbf{P})}{\lambda_{\min}(\mathbf{Q})}$.

The estimation of the convergence time T is optimal when $\mathbf{Q} = \mathbf{I}$, and $\mathbf{I}$ is the unit matrix
